# Naturalistic Developmental Behavioral Interventions: Empirically Validated Treatments for Autism Spectrum Disorder

**DOI:** 10.1007/s10803-015-2407-8

**Published:** 2015-03-04

**Authors:** Laura Schreibman, Geraldine Dawson, Aubyn C. Stahmer, Rebecca Landa, Sally J. Rogers, Gail G. McGee, Connie Kasari, Brooke Ingersoll, Ann P. Kaiser, Yvonne Bruinsma, Erin McNerney, Amy Wetherby, Alycia Halladay

**Affiliations:** 1 University of California, San Diego, San Diego, CA USA; 2 Duke University School of Medicine, Durham, NC USA; 3 M.I.N.D. Institute, University of California, Davis, Medical Center, Sacramento, CA USA; 4 Center for Autism, Kennedy Krieger Institute, Baltimore, MD USA; 5 Emory University, Atlanta, GA USA; 6 University of California, Los Angeles, Los Angeles, CA USA; 7 Michigan State University, East Lansing, MI USA; 8 Vanderbilt University, Nashville, TN USA; 9 IN S.T.E.P.P.S., Irvine, CA USA; 10 Florida State University, Tallahassee, FL USA; 11 Autism Science Foundation, New York, NY USA

**Keywords:** Early intervention, Naturalistic, Developmental, Behavioral

## Abstract

Earlier autism diagnosis, the importance of early intervention, and development of specific interventions for young children have contributed to the emergence of similar, empirically supported, autism interventions that represent the merging of applied behavioral and developmental sciences. “Naturalistic Developmental Behavioral Interventions (NDBI)” are implemented in natural settings, involve shared control between child and therapist, utilize natural contingencies, and use a variety of behavioral strategies to teach developmentally appropriate and prerequisite skills. We describe the development of NDBIs, their theoretical bases, empirical support, requisite characteristics, common features, and suggest future research needs. We wish to bring parsimony to a field that includes interventions with different names but common features thus improving understanding and choice-making among families, service providers and referring agencies.

Our increased ability to identify and diagnose children with autism spectrum disorders (ASD) at ever earlier ages provides us with both an opportunity and a challenge. The last 20 years of research have demonstrated both methods for identifying ASD in even younger children, and also methods for improving outcomes of those children through specific early intervention practices. These advances now allow us the opportunity to begin intervention much earlier in life. Our challenge, however, is to design and adapt our interventions to very young children in order to achieve optimal outcomes (Dawson 2008). While there is a substantial research base supporting the effectiveness of behavioral interventions across the lifespan of ASD, empirical data on the efficacy of interventions that meet the needs of toddlers with ASD have begun to emerge only recently. Most of these studies of toddler intervention are based on behavioral^1^ interventions that utilize more “naturalistic” approaches and developmental orientations than traditional applied behavior analytic (ABA)-based behavioral interventions, such as those beginning with highly structured teaching with older children. For example, the more recently developed toddler interventions often are delivered in naturalistic and interactive social contexts, such as play and daily routines, from the beginning, and involve child-directed teaching strategies, such as use of child-preferred materials. These interventions are based on empirically-based intervention methods derived from both the principles of behavioral learning and developmental sciences. In this paper, we refer to these approaches as “Naturalistic Developmental Behavioral Interventions (NDBI)” to best reflect the dual contributions of these fields.

While other publications have provided accounts of NDBIs (e.g., Prizant and Wetherby 2005), the present paper brought together a group of autism researchers representing a range of views and diverse disciplines in order to develop a consensus statement regarding the empirical and theoretical bases of NDBIs. Our goal was to describe the influences of both behavioral psychology and developmental science on the evolution of early intervention for ASD and their subsequent convergence in the development of effective, evidence-based NDBIs. In an effort to understand and clarify the various NDBIs available for young children with ASD, this paper examines the historical context in which they have been developed, common characteristics of established, evidence-based NDBIs, and requisite features of NDBIs. Issues related to implementation and future research directions are also discussed.

## Historical Context

It is remarkable to consider that, prior to the early 1960s, many believed that children with ASD were unlikely to respond to treatment. The early work of Charles Ferster and Marian DeMyer (Ferster and DeMyer 1961, 1962) demonstrated that children with autism could indeed acquire new skills via an operant discrimination paradigm. During the 1960s and 70s, the study of operant learning treatment approaches for autism increased (Hingtgen et al. 1967; Leff 1968; Lovaas et al. 1974; Mazuryk et al. 1978). Early work in this area demonstrated the effectiveness of operant methodology to teach a variety of skills: language (Lovaas et al. 1966; Risley and Wolf 1967), social (Odom and Strain 1986; Ragland et al. 1978; Strain et al. 1979), play (Lewis and Boucher 1988; Lifter et al. 1993; Stahmer 1995), self-help (Ayllon and Azrin 1968; Baker 1984), and academic skills (McGee and McCoy 1981) as well as to reduce the occurrence of “interfering” or challenging behaviors (Carr and Durand 1985; Schreibman and Carr 1978). Even during these early years, parents were taught how to use strategies based on these principles of learning to improve their children’s behavior at home (Berkowitz and Graziano 1972). This work reflected the new field of “applied behavior analysis (ABA),” which is the science of understanding how changes in the environment affect human behavior. Ivar Lovaas, the main pioneer of the application of learning principles to children with autism, and other investigators believed these children were largely unsuccessful in learning skills from the natural environment and thus the environment should involve simplified instruction and potent reinforcers. Then the focus would shift to generalizing these skills. (Lovaas 2003). The publication of Lovaas’ (1987) autism treatment study, demonstrating significant gains in IQ and success in typical school placements caused both disbelief and, eventually, a paradigm shift in expectations; massive improvements and even “recovery” for almost half of children treated may be a real outcome of excellent treatment provided early enough in children’s development and with enough intensity (i.e., up to 25–40 h per week for several years). This publication, and subsequent studies demonstrating efficacy of early intervention led to two main trends in the provision of autism early intervention.

First, parents began advocating for their children to receive early intensive behavioral intervention, which led to changes in educational policy and, more recently, insurance reform that increased availability and funding for early intervention. Second, discrete trial training (DTT), the behavioral intervention approach used in the 1987 Lovaas study, became increasingly popular. Briefly, DTT involves one system of implementation of operant methodology in which skills are broken down into separate components and taught one at a time in discrete trials, until the desired behavior is acquired. Ironically, as DTT was becoming increasingly popular with parents of children with autism, by the mid- to late-1980s autism intervention research showed that highly structured interventions such as DTT, while effective in teaching skills, (Schreibman 2005) sometimes led to (1) child failure to generalize newly learned skills across multiple environments and circumstances, (2) the presence of escape/avoidance challenging behaviors, (3) lack of spontaneity and (4) overdependence on prompts. The common approach within DTT to first teach response topographies (e.g., imitation of a word) and only later to teach the meaning of the behavior (e.g., teaching a referent for the sound they are making) potentially contributed to some of the limitations mentioned above. These limitations, plus the success of behavior analysts such as Lovaas, encouraged investigators from varied disciplines to focus their efforts in improving and expanding autism interventions. Advances in the developmental sciences, particularly those pertaining to factors associated with learning, have set the stage for advancing early intervention methodologies beyond DTT.

## Early Developmental Perspectives That Informed Autism Research

Concurrent with intervention research occurring in the area of ABA during the 1980s and 1990s, there was also an explosion of new research on infant and child development. The result was an emergence of more sophisticated and detailed models of early developmental learning processes involved in communication, language, and social learning. These studies were soon carried out in autism as well, leading to new understanding of the early core social and communicative impairments associated with the disorder (Dawson and Adams 1984; Rogers et al. 1993; Sigman and Ungerer 1984; Sigman and Capps 1997). These insights began to influence the development of new treatment strategies and models for autism. For example, autism interventionists began targeting skills that were key precursors to language development, such as joint attention (Mundy et al. 1990), as well as skills that were pivotal for providing a foundation for learning a wide range of other skills (e.g., imitation, social engagement, Rogers and Lewis 1989). The importance of allowing the child to be an active rather than passive participant in therapy was underscored by research demonstrating that infants are active “hypothesis-testers” who learn by forming and testing predictions on their environments (Saffran et al. 1996). Studies of typical infants and toddlers also emphasized the role of the social relationship as an essential context for developing imitation and the foundations of communication (Rogers and Pennington 1991). These studies demonstrated that learning is facilitated by an affective exchange between the child and therapist. At the same time, other research suggested that children with autism have deficits in affective sharing and social motivation (Dawson et al. 1990; Kasari et al. 1990). Thus, interventionists began incorporating strategies to promote affective engagement (e.g., Prizant et al. 2003; Rogers and DiLalla 1991).

As developmental science began to focus on atypical as well as typical learning and growth trajectories, a corresponding interest in autism intervention arose in the field across disciplines. It was recognized that often there was discrepancy between the highly-structured teaching strategies used in DTT and the principles of child learning documented by developmental sciences. Another line of studies demonstrated that young children with ASD followed developmental paths that were more similar than different from typically developing children within various developmental domains (Tager-Flusberg et al. 1990; Lifter et al. 1993; Mundy et al. 1987), leading to emphasis on incorporation of developmental principles and sequences in early autism treatment.

## Relevance and Contribution of Developmental Principles to NDBIs

The theoretical underpinnings of the developmental psychology influences in the NDBIs originate from the works of Piaget (1952), Bruner (1978), Vygotsky (1962), Snow (1977), Gibson (1973), and others. This research shows that children learn best when they are engaged as active participants (Kuhl et al. 2003; Gibson 1973; Yurovsky et al. 2013), in developmentally appropriate learning experiences (Bruner 1983; Vygotsky 1962), and in contexts meaningful to the child (Kuhl et al. 2003). Children learn most easily the skills that are just beyond their present knowledge, and follow regular developmental sequences in virtually all developmental domains (Vygotsky 1978; Piaget 1966). Thus, assessing children’s present skill sets and choosing targets that represent the “zone of proximal development” in each domain facilitates learning rates and successes (e.g. Lifter et al. 1993). In the NDBIs, a constructivist approach is taken—children’s learning experiences are strategically designed to actively engage children’s attention, help them connect new experiences with existing knowledge, teach within developmental sequences, and, through systematically increasing complexity of the learning experiences, enable them to discover the regularities in the world around them. Child initiative and spontaneity are fostered and rewarded, further promoting children’s contributions to their own learning in the constructionist tradition.

In addition, developmental psychology research examining environmental factors that promote child social cognition, language learning, and play has been mined to construct interventions for children with autism and other developmental disorders. For example, young children develop their skills in the context of affectively rich social interactions involving play with both people and objects. Identical information delivered outside the context of an affectively engaged social exchange does not result in the same degree or depth of learning (Kuhl 2007). Interventions anchored in developmental principles aim to effectively and efficiently promote learning characterized by cross-domain integration of social, language, and cognitive skills and knowledge. For children with ASD, this is viewed as particularly important because of their core difficulties in these areas (Tsatsanis and Powell 2014). Application of developmentally-informed principles in early intervention is also designed to promote generalization throughout the intervention process as well as socially appropriate and functional use of new skills and knowledge. Everyday routines present particularly rich learning contexts for children (Ratner and Bruner 1978) and teaching within these assures that children’s new learning is incorporated into everyday life and supports children’s adaptive functioning in natural contexts and environments

## Trend Towards Increasingly Naturalistic Behavioral Interventions

Efforts to improve the effectiveness of DTT procedures quickly led to incorporation of new techniques for increasing children’s motivation and performance—techniques that would ultimately prove quite compatible with the models of early learning processes being developed in the developmental sciences. Such techniques included varying teaching stimuli (Dunlap and Koegel 1980), alternative prompting strategies (Schreibman et al. 1982), use of child-preferred activities Koegel et al. (1987a), use of incidental teaching strategies (McGee 2005) and consideration of developmental prerequisites (Dawson and Galpert 1986, 1990; Lewy and Dawson 1992; Kasari et al. 2006, Rogers and Lewis 1989). These newer approaches used natural rather than artificial (arbitrary response-reward contingencies) rewards (Koegel and Williams 1980), child-preferred materials (McGee et al. 1991), reinforcement of approximations and communicative attempts, and treatment delivery in more naturalistic and developmentally sensitive contexts (McGee et al. 2000).

Despite apparent differences, the highly structured teaching approaches (e.g., Verbal Behavior; Sundberg and Michael 2001) and naturalistic teaching approaches are all firmly grounded in principles and the science of learning (McGee 2005). All fully meet criteria as ABA techniques including (1) intervention protocols that are composed of operant teaching techniques; (2) intervention goals that are socially significant; and (3) intervention results are analyzed objectively by assessing a child’s progress before, during and after the intervention (Baer et al. 1968). In addition, both naturalistic and highly structured teaching approaches are enhanced by research in areas of experimental analysis of behavior, such as shaping, fading, discrimination training, and errorless learning (McGee et al. 1986).

Early applications of NDBI in early autism found that generalization improved substantially as a result of teaching in the context of naturally occurring activities (Carr and Kologinsky 1983; McGee et al. 1983). Procedural comparisons subsequently showed that teaching in the context of natural environments, in which the cues were continually changing, yielded better generalization and decreased the need to directly teach each skill in multiple and varied situations (McGee et al. 1985). Related findings showed that children with autism learned more rapidly when there was a natural, rather than an arbitrary, relationship between a response and the reward for using that response (e.g., saying “car” and receiving a car to play with versus saying “car” and receiving a piece of candy for correct labeling of the car); such research contributed to development of two widely known naturalistic behavioral interventions, Incidental Teaching (McGee et al. 1983) and pivotal response training (PRT; Koegel et al. 1987b; Koegel and Koegel 2006; Laski et al. 1988; Schreibman and Koegel 2005).

Naturalistic behavioral interventions to autism have demonstrated special promise when children are very young and are less likely to have established patterns of maladaptive behavior. In addition to previously referenced generalization gains, the following procedural benefits have been associated with the use of these interventions in young children with ASD: (a) reduced dependence upon prompts (McGee et al. 1983), (b) more natural-sounding language (McGee and Daly 2007), (c) efficiency advantage of teaching language form with meaning (McGee et al. 1985) and (d) habituation to everyday distractions present in the real world (McGee 2005). Research also has demonstrated that naturalistic interventions are conducive to promoting social development in that they typically involve interactive exchanges between the child and an adult or typically developing peer (Morrier et al. 2009). Further these are “family friendly” approaches that tend to increase both the quantity and quality of early learning experiences. Parents can readily implement these strategies in their natural environments and during ongoing activities such as meals, bath time, and visiting a park (McGee 2005; Schreibman and Koegel 2005).

Interest in naturalistic behavioral methodologies occurred at about the same time that autism researchers were identifying early signs of autism in toddlers and discovering the benefit of providing interventions to children with autism at younger ages (Fenske et al. 2001; Fenske et al. 1985; Lovaas 1987; McGee et al. 1999). Finally, as we shall describe in more detail below, naturalistic behavioral interventions began to consider a child’s developmental readiness (including both developmental and chronological age) when choosing learning goals and developing behavior plans (Feldman et al. 1994; McGee et al. 1997). For example, research supported the idea that teaching new skills, such as play actions, to children with autism at their developmental, rather than their chronological age leads to improved acquisition, generalization and maintenance of new skills (Lifter et al. 1993).

Naturalistic behavioral interventions provided a different perspective and approach to handling unwanted and challenging behaviors, which led to a diminishment of their frequency (McGee and Daly 1998). With a greater focus on development, some challenging behaviors, such as tantrums, were viewed as normative for young children with or without autism. Many toddlers are expected to have tantrums and challenging behaviors; using interventions that had begun to take into account developmental level proved to be successful in helping children to learn to regulate their own behaviors (much as young typically developing children do). Further, when children participate in naturalistic interventions, they receive instruction where they want to be, doing what they want to be doing. The process of securing a child’s attention is essentially built into naturalistic strategies because the teaching materials are toys/items/events that are desired by the child. Importantly, and not surprisingly, naturalistic strategies are associated with reduced escape- and avoidance-motivated behavior (Koegel et al. 1987a, b).

While we are focusing here on naturalistic behavioral interventions it is imperative to emphasize that although an important impetus for the development of these interventions was addressing some limitations of highly-structured behavioral interventions, these new behavioral approaches likely would not exist without the prior successful highly-structured interventions such as DTT. In addition we acknowledge that while massed trials may be used in the initial stages of DTT intervention, in later stages DTT researchers focus on reducing the massed trial aspect of treatment and incorporate other strategies as well. Many researchers and clinicians using contemporary DTT-based interventions now incorporate NDBI approaches as part of a continuum of teaching approaches used with individual learners. So for many DTT investigators and practitioners massed trial may be only a small part of the overall approach and in fact some eschew any massed trials at all (Green 2001; Grow and LeBlanc 2013). From a NDBI standpoint beginning with highly structured, decontextualized programming used in typical DTT-based intervention might not be required. Perhaps children with autism actually do learn from the natural environment when learning opportunities are structured appropriately, especially if they are taught key skills for learning in that context (e.g., joint attention). It is also important to acknowledge that massed trial DTT teaching remains the approach of choice for certain skills at certain times, for all human learners, and it remains an important tool in the autism intervention toolbox (Jobin 2012). Furthermore, it is likely that some children may learn more quickly using a more structured approach, such as DTT, whereas other may flourish using a NDBI approach. As a controlled randomized trial has yet to be conducted with a head-to-head comparison of NDBI versus DTT, an important research goal involves learning for whom, and for what skills, naturalistic versus highly structured teaching is most helpful.

## Integration of Developmental Principles and Applied Behavior Analysis

Historically, behavioral and developmental research reflected two fields that operated from diverse and somewhat distinct perspectives, theories, and methodologies, with different implications for clinical practice. Behavioral scientists often were less attuned to the rich body of information on typical child development when formulating behavioral interventions, and developmental researchers often were less attuned to the learning science principles crucial for fostering rapid skill building. As both fields matured and were challenged by the need to intervene in developmental problems earlier and earlier, it became apparent that interventions needed to take into account both what had been learned about early child development, and how infants and toddlers learn when choosing treatment targets and teaching strategies for young children. For example, research showed that teaching foundational skills such as joint attention, gesture, and shared affect facilitated the later acquisition of language (Kasari et al. 2008). Thus, an appropriate treatment goal for language development is to focus on these foundational skills rather than trying to teach language via verbal imitation alone. Interventions began to emerge that were mutually informed by developmental and behavioral principles, demonstrating that these two fields could be integrated and that interventions could incorporate the strengths offered by each perspective.

## Core Components of NDBI Intervention

Core components of NDBIs fall into three general areas: the nature of the intervention targets; contexts in which the interventions are delivered; and instructional strategies.

### Nature of the Learning Targets

The intervention targets within NDBIs often include the entire range of developmental domains, including cognition, social, language, play, and motor systems (e.g., Dawson et al. 2010; Landa et al. 2011). Furthermore, in contrast to highly-structured teaching approaches, NDBIs emphasize the integration of knowledge and skills across developmental domains and promote generalization of newly learned skills at every phase of the intervention process. In other words, NDBIs reflect a developmental systems approach, in which the goal is to ensure that development of a skill in one domain (e.g., learning a symbol, such as a new word or gesture, in one activity) will be integrated with development of skills in other domains (e.g., using the word or gesture to sustain engagement with another person and in other activities) from the beginning. Thus, in the NDBI approaches, skills are usually not taught discretely or in isolation, but rather in the course of the child’s typical daily interactions, experiences, and routines, with multiple materials and by multiple people. NDBI approaches do not strive primarily to enlarge the child’s behavioral repertoire per se within a skill domain, but rather to provide an infrastructure to support efficient and effective learning involving functional skills used in everyday life, particularly social-communication learning via interactive, meaningful exchanges with others. The core components of learning that support development of a wide range of skills involve such abilities as attending to others, imitating others, sharing emotions and interests via joint attention, sharing a common frame of reference with a partner about an environmental event, engaging in coordinated, reciprocal activities with others, and understanding that meanings are transmitted between people via gestures, sounds, expressions, and words. As this core is established, development of the ability to comprehend and produce an ever-increasing repertoire of new and more complex forms across all developmental domains (e.g., words, gestures, phrases, play acts and sequences) is facilitated.

To build a strong learning infrastructure, intervention targets focus on developing knowledge and abilities that have been shown to be precursors of certain developmental achievements, or that are known to enhance these achievements. Two examples that are particularly relevant to young children with autism are joint attention and imitation. Joint attention refers to the use of gestures, gaze, and/or language for the purposes of sharing information about objects/events with other people. For example, joint attention occurs when the child points to something in their environment for the purpose of showing the object to another person or commenting on an event. The development of joint attention gestures, particularly initiating joint attention, has been associated with better language skills in typical children and in children with ASD (Mundy et al. 1990; and others).

Imitation is a critical tool for learning and for social acceptance. Even before speech develops, children’s ability to imitate offers them a platform for engagement with others and a means for learning from others. The ability to imitate provides children with the opportunity to take a turn in a social interaction and share others’ topics. It also enables children to synchronize their experiences with others, thereby experiencing another person’s state. In effect, imitation creates an opportunity for children to develop awareness that they are like others, which is likely to be linked to development of theory of mind (Meltzoff and Gopnik 1993). As children become attuned to others’ actions and relate those actions to themselves, they begin to be able learn more effectively from observing others. That is, they can learn by watching others, without having to experience every cause-effect relation themselves, thus accelerating their learning and allowing human cultural inventions like language, tools, symbols, games, and all kinds of motor and artistic skills, among many others, to be passed on across the generations. Interventions for children with ASD have shown that they can learn to imitate in socially engaged ways (Landa et al. 2011; Ingersoll 2010) within NDBIs.

### Nature of the Learning Contexts

The empirical literature has documented that children’s experiences affect their neurobiological development (Dawson et al. 2012; Knudsen 2004) and that experiences have a cascading effect on development (e.g. Thelen and Smith 1994). The contexts within which early learning occurs need to allow children to experience the natural contingencies of their own behavior (Gibson 1973). Increasing evidence is emerging that learning is enhanced when it is embedded in activities that contain emotionally meaningful social interactions compared to situations in which instruction occurs without meaningful social engagement (Topál et al. 2008). Spelke and colleagues argue that providing children the opportunity to learn within a socially engaged context sets the stage for children to learn about the social landscape around them (Spelke et al. 2013). Within NDBIs, this is often accomplished through establishing adult-child engagement activities that transform into motivating play routines or familiar daily life routines. Contingency-based skill building can be more effective in this engaged context. Thus, specific characteristics of learning contexts, including the activities being used, the quality of relationship between child and adult, and emotional valence of the activity and interaction for the child facilitate the learning and generalization of newly developing skills.

### Nature of the Development-Enhancing Strategies

The development-enhancing strategies used within the NDBIs (see specific strategies below) work together to support high levels of success inside ecologically valid contexts, routines and materials within them. The motivating activities created as part of the intervention process begin as very simple action sequences, where contingencies between the child’s behavior and a rewarding experience are highly predictable and salient. For example, a playful routine involving tickles when putting on the child’s shirt during a dressing routine may be expanded to include receptive language skill building as well as social commenting. The child may be instructed to follow directions to ‘get your shirt’, where he must select the shirt from an array of other clothing, then encouraged to show his ‘red shirt’ to his sister by saying “Look! My red shirt!,” being prompted as necessary. By incorporating behavioral strategies such as modeling, shaping, chaining, prompting, and differential reinforcement, the adult supports the child to expand language, the complexity of the play acts, the social demands, or the number of action sequences within the routine as the child masters simpler levels. With increased duration and quality of children’s engagement, adults infuse the engagement with increasing numbers and types of symbols, and symbol combinations (e.g., non-linguistic via play and linguistic via speech). Within these supported joint activities, the interventionist systematically expands children’s reciprocity, communication, social, and play skills as well as scaffolding increasingly age appropriate cognitive, motor, and adaptive skills. The rewarding value of these child-centered, everyday activities heightens children’s motivation, and as noted above, maladaptive behaviors often wane as they are replaced by carefully chosen teaching targets that represent more socially conventional behaviors.

## Examples of Naturalistic Developmental Behavioral Interventions

It has been very encouraging that multiple clinical research laboratories throughout the country have independently established NDBIs. This suggests that multiple researchers were drawing the same conclusions regarding important changes necessary in behavioral intervention for children with ASD. Since they have been developed independently they go by several different names. Examples include Incidental Teaching (IT; Hart and Risley 1968, 1975; McGee et al. 1999), pivotal response training (PRT; Koegel and Koegel 2006; Koegel et al. 1989; Schreibman and Koegel 2005), the Early Start Denver Model (ESDM; Dawson et al. 2010; Dawson et al. 2012; Rogers and Dawson 2010; Rogers et al. 2012), enhanced milieu teaching (EMT; Kaiser and Hester 1994), reciprocal imitation training (RIT; Ingersoll 2010; Ingersoll and Schreibman 2006), Project ImPACT (Improving Parents As Communication Teachers (Ingersoll and Wainer 2013a, b), Joint Attention Symbolic Play Engagement and Regulation (JASPER; Kaale et al. 2012, 2014; Kasari et al. 2006, 2008, 2010, 2014a, b), Social Communication/Emotional Regulation/Transactional Support (SCERTS; Prizant et al. 2003) and Early Achievements (Landa et al. 2011; Landa and Kalb 2012). This list certainly is not exhaustive, nor is it meant to be, but includes some of the most researched models. Each of these intervention packages has its own specific features and there are differences among them. One main difference is that some are focused interventions; addressing a specific behavioral area such as social-communication (e.g., JASPER, RIT) others are comprehensive interventions in that they target a wider array of functioning, including communication, cognitive, motor, and adaptive behavior (e.g., ESDM). Despite some differences, their commonalities are the emphasis here. Many of these intervention packages have been tested using randomized controlled clinical trials; however there have been no published large scale RCTs that have compared DTT versus NDBI interventions or two different NDBIs, although such trials are underway.

## Common Features of NDBIs

While, there are some procedural and technical differences between existing NDBIs, several evidence-based features stand out as common threads across these interventions.

### Three Part Contingency

As noted above, evidence-based NDBIs are *based upon well*-*established principles of applied behavior analysis.* Thus, they represent ABA treatment. All of the NDBIs utilize a three-part contingency (antecedent-response-consequence) to help the child understand when to respond and to provide feedback to the child. However, the emphasis on contingency components may vary across interventions. For example, some interventions provide a clear antecedent in order to gain a specific child response, while other interventions prioritize environmental arrangement to facilitate initiation and responding behaviors from the child. Some interventions clearly specify contingent reinforcement as a component while others use the strategy without specifying it in behavioral terms.

### Manualized Practice

Each of the NDBIs reviewed here has *clear procedures carefully described in their respective intervention manual*s. Accurate implementation of an intervention requires clearly stated procedures (Durlak and DuPre 2008; Fixsen et al. 2005; Greenberg et al. 2005). Manualization helps with training and consistency of treatment implementation among treatment providers (professionals, parents, etc.). Some interventions have publicly available, published manuals, while others use manualized information available primarily in a research setting. Of course, manualization and clearly specified procedures are necessary, but not sufficient, for appropriate and effective implementation of an evidence-based intervention. Additional training, including coaching and feedback, is necessary to facilitate accurate use of an intervention (Bush 1984; Cornett and Knight 2009).

### Fidelity of Implementation Criteria

In order to determine whether an intervention is effective there must be some type of definition of its correct usage. Therefore, each of the interventions examined here has *fidelity of implementation assessments* available to ensure integrity of treatment implementation. Fidelity of implementation is the degree to which a treatment is implemented as it is supposed to be (e.g., Gresham 1989; Rabin et al. 2008; Schoenwald et al. 2011). Some also include assessments of therapist competence as part of the fidelity measurement (the level of skill and judgment used in executing the treatment (Schoenwald et al. 2011). Fidelity of implementation is likely a potential mediating variable affecting child outcomes, with higher fidelity of implementation of an effective procedure resulting in better outcomes (Durlak and DuPre 2008; Gresham et al. 2000; Stahmer and Gist 2001). The lack of reporting (and therefore, the presumable lack of actual measurement of implementation) limits the conclusions that can be drawn regarding the relation between child outcomes and the specific treatment provided. It is a demonstration of the current expectations of new intervention approaches that they include methods of measuring treatment fidelity of implementation.

### Individualized Treatment Goals

All of the interventions reviewed use some developmentally-based strategies and use developmental sequences to guide goal development that is individualized to each child. Some interventions do this through the use of a specific, developmental assessment and curriculum. For example, in the ESDM (Rogers and Dawson 2010), SCERTS (Prizant et al. 2003) and Early Achievements (Landa et al. 2011) models, a treatment-specific curriculum assessment with measureable behaviors across developmental domains is completed to guide the development of specific goals for each child. Similar approaches are used in targeted treatments where the selection of a specific teaching target is individualized per the sequenced target guidelines in the intervention model (e.g., play and joint attention targets in JASPER). In nearly all NDBIs, goals are typically developed with the use of standardized assessment, observation and developmental checklists, which help guide the clinician in choosing developmentally appropriate treatment goals across domains and teaching targets.

### Ongoing Measurement of Progress

Effective practices must be systematically and objectively verified through data collected (Simpson 2005a, b), and the research-based NBDIs provide methods for systematic data collection on child progress in order to track child progress. In addition, data are collected to examine the success of the intervention as a whole. Data collection methods may include trial-by-trial recording of children’s response to each opportunity, interval recording of child progress during a session, probes of specific behaviors, and use of curriculum-based assessments to examine progress at specific time periods (e.g., monthly or quarterly). Data collection is a critical aspect to any approach based in ABA. The method of data collection should be linked to child goals and then used to adapt the intervention to the specific needs of the child and family.

### Child-Initiated Teaching Episodes

These are also referred to as following the child’s lead or interest, or child choice. This strategy involves the presentation of an instruction or opportunity to respond within the context of a child-chosen or child-preferred activity or familiar routine. The child indicates an interest in an activity or engages in a familiar routine and the adult then presents a teaching opportunity within that activity. The goal of child-initiated teaching episodes is to increase the child’s motivation for participation and to use the child’s achievement of his or her goal as the positive consequence for the child’s use of the target skill set up by the adult. The degree to which the child must initiate the teaching episode differs across interventions. For example, Incidental Teaching requires the child to initiate an interaction (make a communication bid) prior to presenting a prompt for an elaborated response. Other approaches (PRT, ESDM, Project ImPACT, Early Achievements) may also present a stimulus to gain child approach behavior or child attention, and then prompt the child for a target skill, and RIT presents an instructional cue (play model) based on the child’s current attentional focus every minute on average; such follow-in directives are associated with language development in young minimally verbal children with autism (Haebig et al. 2013). Most approaches use a blend of these.

### Environmental Arrangement

This set of techniques involves setting up the environment so that the child must initiate or interact with the adult in order to obtain a desired outcome, such as access to preferred materials or participation in preferred routine. Environmental arrangement also refers to how the adult structures the environment to facilitate child initiation of skills and learning of new target skills. Across interventions, this strategy might also be referred to as communicative temptations or controlling access and is used to encourage the child to initiate an interaction with the adult or to allow the adult to deliver the desired object contingent on the child’s performance of the learning target. The use of specific environmental arrangement strategies varies across interventions, and may include controlling access to materials of interest, playful obstruction, expectant waiting, violating a routine, using materials that require assistance, and placing desired items in sight but out of reach. It may also specify adult and child positioning, and material choice based on child developmental level and teaching targets (e.g., JASPER). Several interventions delineate specific environmental arrangement strategies, while others simply require that the adult gain the child’s attention typically using some form of social orienting cue alone or in combination with controlling access or blocking play prior to presenting a prompt.

### Natural Reinforcement and Related Methods for Enhancing Motivation of the Child

Natural reinforcement is reinforcement that is intrinsic to the child’s goal rather than unrelated to the child’s goal (external or extrinsic to the theme or content of the activity or interaction). For example, imitation of a symbolic play act with a preferred toy would be reinforced by the child’s continued access to the toy and freedom to play as the child wishes (generally paired with social attention). This is in contrast to more traditional behavioral strategies that involve having a child complete a task (e.g., push the car) and then receive an unrelated reward such as a token, break, different toy or food. A procedure related to natural reinforcement is the use of loose reinforcement contingencies, also referred to as *loose shaping* or *reinforcing attempts*. The goal of this is to keep the child’s motivation high and to reinforce “trying,” or initiating, while teaching novel behaviors (Koegel et al. 1988), though there is variation across approaches in terms of how closely the child’s performance matches the target in order to receive the reinforcer. An additional method for enhancing motivation involves interspersing easier (already mastered) tasks and more difficult (target learning skills) tasks. This technique requires the adult to elicit some skills (e.g., word production, play action) that the child is already able to use independently along with skills that child has not yet mastered. For example, a child who uses primarily single words would receive a model involving a two-word phrase (acquisition task) during some teaching interactions and single words (maintenance task) in other teaching interactions to request desired actions during play. The child’s responses would be reinforced for copying the model in both types of trials. The goal of this strategy is to increase the child’s motivation, decrease frustration due to failure, and to maintain learned skills through the presentation of mastered skills, while helping the child acquire more advanced skills. Also, varying the degree of complexity of targeted skills helps to keep the children’s language and play interactions more natural, as typically developing children use varying levels of speech (“I would like an apple please, Mom.” and “More apple!”) and play (sometimes engaging in elaborate sociodramatic schemes and other times tossing a ball with the interventionist). Several NDBIs specifically require this technique, while the other interventions achieve this through loose shaping (by reinforcing a mastered or maintenance skill as an attempt).

### Use of Prompting and Prompt Fading

Prompting, also referred to as scaffolding or cuing, involves inserting a cue (verbal, visual, or physical) between the instruction or discriminative stimulus (S^d^) and the target behavior in order to elicit a desired response and thereby create the context for delivering the reinforcer. The goal of prompting is to support behaviors currently outside of the child’s repertoire or not yet under the control of the S^d^ so they can occur and be reinforced, thus leading to an increase in those behaviors. Some of the NDBIs delineate specific prompt strategies for the adult to use while other interventions are less specific about the types of prompts used. However, all NDBIs require the systematic use of adult prompts to promote new skills and systematic delivery of contingent reinforcers, which, along with systematic ongoing data collection, defines primary differences between NDBIs and developmental interventions that do not incorporate ABA principles.

### Balanced Turns Within Object or Social Play Routines

This strategy has also been referred to as shared control, turn-taking, balanced turns, or reciprocal interactions. The goal of this technique is to increase social reciprocity, maintenance of interactions, and turn-taking with materials, as well as to create opportunities for the adult to control access to the materials. Because therapist turn-taking focuses on supporting the back-and-forth interactional structure that is a primary mechanism of early learning (Harris and Waugh 2002), its inclusion in ASD interventions is intuitively appealing. However, despite the widespread incorporation, unlike other strategies of NDBIs, there has been limited empirical investigation of the practice in isolation. A recent pilot exploration of turn taking in the context of PRT suggests that the specific implementation of the strategy may affect behaviors (e.g., requesting, commenting, toy play) differentially depending on a child’s developmental level (Rieth et al. 2013). For some NBDIs (ESDM, PRT), balanced turns is considered a key feature of the intervention, while for other interventions balanced turns occur within the context of building longer interactions.

### Modeling

Modeling involves adult demonstration of a behavior that follows the child’s focus of interest and often demonstrates the target skill the child is to display. Modeling is used to teach target skills from most domains: language, imitation, social, play, cognitive, motor skills, in addition to some self-care skills. Modeling is often used as a specific prompt strategy, such that the child is expected to imitate the modeled action or language, as in RIT and ESDM. Importantly, the modeled behavior is carefully chosen with developmental considerations in mind, such as modeling behaviors slightly more advanced than the child’s current developmental abilities.

### Adult Imitation of the Child’s Language, Play, or Body Movements

This technique is referred to as contingent imitation, mirroring, or reciprocal imitation and is used to increase the child’s responsivity and attention to adult, imitation of adult, and continuation of the interaction. Research indicates children with ASD (and typical development) respond with increased attentiveness to the adult partner when being systematically imitated (Dawson and Adams 1984). Different NDBIs vary in the degree to which imitating the child is a central feature of the intervention. For example, in RIT, JASPER and ESDM, it is a central treatment component, but other interventions place less emphasis on it and some may not consider it a key component.

### Broadening the Attentional Focus of the Child

Early research pointed to a specific attentional deficit, stimulus overselectivity, that characterized the responding of many children with autism (e.g., Lovaas et al. 1971). Stimulus overselectivity refers to the phenomenon wherein the child’s behavior comes under the control of a range of stimuli that is too limited and/or stimuli that may be irrelevant. (For example, a little girl might recognize her father only by his glasses but when his glasses are removed, she no longer recognizes him.) It is easy to see how such overly restricted attention would interfere with learning. Research has demonstrated that overselectivity is partly a developmental phenomenon (Ploog 2010; Reed et al. 2013) and thus may not be as specific to ASD as once was believed. Overselectivity can be modified in many children (e.g., Koegel and Schreibman 1977) and teaching with multiple and varied stimuli seems to be key. NDBIs, with their emphasis on teaching in natural and varied settings, with a range of real life materials, may likely help broaden, or normalize, the child’s attentional focus (Dawson et al. 2012; Rieth et al. 2014).

## Support for NDBIs as Validated, Evidence Based Treatments

Recent reviews of efficacious intervention models have included NDBIs (Dawson and Bernier 2013; Dawson and Burner 2011; Maglione et al. 2012; National Standards Project 2009; Odom et al. 2010; Vismara and Rogers 2010). For children with ASD, researchers recommend interventions that include parent education (for generalization and additional learning opportunities), start as early as possible, and blend behavioral and developmental strategies to address core issues such as engagement and joint attention while systematically improving specific communication, cognitive and other skills (Wallace and Rogers 2010). Several controlled, single-subject and quasi-experimental studies (Ingersoll and Dvortcsak 2006; Ingersoll et al. 2005; Stahmer et al. 2011a; Stahmer and Ingersoll 2004) and recent randomized clinical trials (Dawson et al. 2010; Kasari et al. 2006; Yoder and Stone 2006; Wetherby et al. 2014) suggest that including a parent coaching component accelerates developmental progress in ASD. Indeed, in a study involving the largest RCT of young children using a NDBI approach (Green et al. 2010), parent synchronization to child activity mediated child outcomes). These studies suggest that an integration of developmental and behavioral methodologies represent state-of-the-art treatment for serving the youngest children with ASD (e.g., Dawson et al. 2010; Landa et al. 2011; Stahmer et al. 2011a, b; Rogers et al. 2014).

## Future Research Directions on NDBIs with Children with ASD

NDBIs for children with ASD have been developed systematically through research studies using both single case and group experimental methods as discussed throughout this paper. NDBIs have a combined developmental and behavioral analytic conceptual foundation and strong empirical foundation. In the course of the last three decades, research on naturalistic interventions for children with ASD has established that these strategies can be implemented with high fidelity in clinics, homes and schools and can result in consistent positive outcomes, especially for communication, language and social behavior (e.g., Kaale et al. 2012; Kasari et al. 2014a, b; Wetherby et al. 2014). The majority of early studies implemented single case designs (e.g., Koegel et al. 1987a, b, 1998; Laski et al. 1988, Pierce and Schreibman 1995, Stahmer 1999). In the second generation of studies on naturalistic teaching, researchers have tested the effects of these procedures in randomized trials with increasingly more sophisticated designs (Dawson et al. 2010; Dawson et al. 2012; Kasari et al. 2006, 2010, 2014; Landa et al. 2011; Wetherby et al. 2014). In several instances, two or more naturalistic teaching methods have been integrated into a comprehensive intervention protocol (e.g., ESDM is based on Denver Model and PRT). Experimental applications of naturalistic teaching have demonstrated efficacy when including parents, therapists, teachers, and others as interventionists (e.g., EMT, PRT, JASPER, SCERTS). While studies to date provide considerable empirical support for the effectiveness of naturalistic interventions, there is a need for continued research to *refine the active ingredients of the procedures, to test the long*-*term effects of the procedures, and to increase the efficiency and effectiveness of naturalistic interventions*. In particular, larger scale research studies are needed that include measures of meaningful, functional outcomes across contexts and over time and which examine the range of child responses to treatment.

Six areas of research are recommended to advance the development of NDBIs. These areas represent a continuum of related topics rather than six discrete areas of research endeavor. In keeping with the history of research on naturalistic teaching, the next generation of studies on NBDI needs to provide:

1. Increased emphasis on larger scale and more contemporary RCT designs that can address moderators and mediators and efficiency of treatments.

The first generation of randomized trials of naturalistic interventions targeted young children with the goal of preventing or ameliorating the early social and communicative indicators of autism, and/or determining the efficacy of a particular treatment approach. Sufficient sample sizes for testing the effects of the NDBIs with children who are older or who have not responded to other types of early intervention are an important need (see e.g., Kasari et al. 2014a, b application of a SMART trial). Examination of mediators and moderators of treatment are also a logical next step in developing more targeted treatments. As an example, Sherer and Schreibman (2005) identified a behavioral profile correlated with outcome of children with ASD receiving one of the NDBIs, PRT. It is essential that future studies expand the description of participants in randomized trials in terms of both their autism diagnostic status and the extent of their delays in expressive and receptive language and social behavior in natural contexts. Further, it is important that the dosage of treatments (total hours of treatment) be reported in order to make judgments about the relative efficiency of treatment. Assessments at multiple time points within the treatment should be used to gauge the outcomes associated with specific dosages and time in intervention.

2. Measurement of intervention outcomes that represent meaningful change.

In the context of a history of studies demonstrating changes in IQ but relatively weaker changes in developmental outcomes such as core ASD symptoms and limited measurement of long term social functioning, it is important to extend measures of outcomes to include both proximal and distal estimates of *functional* changes in child behavior in everyday social contexts (e.g., parent–child interactions at home; interactions with peers in childcare or preschool). While there are studies reporting proximal outcomes (e.g., change within intervention sessions, in probes to untrained partners) few studies report change in everyday environments such as home and childcare settings. Observations of interactions with parents outside of the clinic or research setting and of child engagement, participation and demonstration of social and communication skills in child care or preschool settings are essential and could provide important outcome data for determining if interventions affect children’s everyday functioning (see Kaale et al. 2012; Kaiser, et al. 2014a, b; Lawton and Kasari 2012; Wetherby et al. 2014). Such observations might include relatively straightforward assessments of generalization and maintenance across settings and partners leading to the development of benchmarks that could then be used to set standards for expected impact on children’s functional behavior in everyday settings. However, efficient methods for conducting observational measures in large group studies would facilitate our ability to measure generalization. Additionally, common, standardized measures of social functioning examining changes in core deficits are also needed (Anagnostou et al. 2014).

3. Empirical analysis of the active ingredients within multicomponent interventions.

Naturalistic interventions vary widely in the range of specific strategies included. For example, modeling, balanced turns, natural reinforcement and prompting are common components. The relative contributions of these strategies and the necessity of including several strategies at prescribed levels are typically unknown. In most cases, researchers do not yet have empirical evidence to support the frequency, quality or relative balance of strategies included in treatment packages. These types of dismantling studies also are needed in order to move to the next step of matching specific active ingredients to an individual or dyad (Stahmer et al. 2011a, b). And of course this may vary enormously across children, as the heterogeneity of the population is well known. These data are especially important for moving these interventions into community settings as community adoption is more likely if interventions are easier to implement and methods for adoption in varying settings and for individual children are clearly specified. More information regarding factors affecting efficacy of the interventions will enhance our ability to tailor more effective and efficient interventions at the level of the individual. Studies that establish conceptual and empirical links between active ingredients and both behavioral outcomes and underlying functional brain activity are also needed (Dawson 2008; Sullivan et al. 2014).

4. Understanding the necessary procedural fidelity of individual components within treatments and treatment packages.

Measures of treatment fidelity are essential in both evaluating the quality of evidence supporting the use of naturalistic treatments and in describing the quality indicators and dosage parameters for translating research protocols into practice. These are available for NDBIs, which will facilitate the next generation of research on NDBIs, which must include replication of naturalistic treatment protocols by researchers not associated with the development of the protocol. Additionally, procedures often need to be adapted to fit the community or cultural context. Understanding how the interventions can be modified and individualized for different children and contexts while remaining effective is essential. Both types of research require that the intervention procedures (individual strategies, combinations of strategies, multi-component packages of procedures, procedures for training implementers) and procedures for establishing and assessing fidelity are well-described and accessible to researchers.

5. Developing new methodological approaches to test treatment strategies for improving the outcomes of NDBIs for all children, including children who are slow or poor responders to a specific treatment.

An important step in advancing the evidence for naturalistic treatments will be examining how treatments can be tailored to maximize outcomes for all children with autism, including those children who are initially poor responders to treatment and those who need long-term intervention to maximize their functional outcomes. To accomplish this, three steps are needed; two of these steps are implied in the discussion above. First, existing data should be examined to determine conceptually and empirically the components of treatments that are effective for children who do and do not respond well to a specific intervention. Second, it must be determined which existing treatment components might be combined to produce better outcomes for children who are initially less responsive to a single treatment protocol. The combination of treatment outcomes ideally will be theoretically grounded and supported by preliminary evidence that combinations of treatments, rather than simply increased dosage of a single component, leads to improved outcomes. Finally, the sequence of treatment combinations must be examined and the timing and functional outcome measures for examining early treatment outcomes must be tested. The adaptation of treatments and development of both procedures and heuristics for determining treatment sequencing is an important, but challenging goal.

6. Utilizing innovative methods to implement and sustain research-based NDBIs in the context of community programs serving children with ASD.

Although researchers have made substantial progress in the development and refinement of NDBIs, they are not yet widely delivered in community settings (Hess et al. 2008; Stahmer et al. 2005), thus limiting the range of types of intervention available to children and families. In order to increase choices and alternatives, researchers have called for innovative models of intervention implementation that shift from the traditional, unidirectional models of translating research into practice toward a more reciprocal, interactive effort between researchers and providers (Bondy and Brownell 2004; Meline and Paradiso 2003; Weisz et al. 2004). Implementation research in other fields indicates that there must be a fit between an intervention and the services system, the providers and the families. NDBIs may be an excellent match for public intervention systems due to their focus on early child development and the naturalistic strategies that are required by early intervention legislation. Challenges such as the complexity of the interventions, cost of high intensity implementation, and demands of training and ongoing support and monitoring (especially in low-resource areas) must be addressed to ensure a fit between these efficacious interventions and community care. Innovative research designs that allow for examination of effectiveness in community programs and examine external validity of these methods are needed (Green and Nasser 2012) to further our understanding of how to ensure that high quality interventions reach a majority of children.

## Conclusion

The field of autism early intervention has changed dramatically in the last 30 years. Since the development of the first empirically-validated and highly-structured ABA interventions that changed the lives of children with autism, continued research has expanded these efforts by moving towards more naturalistic interventions that integrate principles identified by developmental science with ABA principles. Whereas behavioral and developmental research and treatment in ASD initially proceeded separately, the increasing emphasis and evidence on autism intervention during the early childhood period have brought these fields together. These NDBIs represent the integration of ABA and developmental science and they not only allow us to achieve more substantial and accelerated child learning and behavior change, but they are particularly well suited to the infant and toddler autism population now being served.

The various NDBIs share essential features, including implementing intervention in the context of naturally-occurring social activities within natural environments. All are more child-directed than previous ABA approaches, involve intrinsic rewards for learning and participating, allow for sampling a wide range of antecedent stimuli and acceptable responses during the teaching interaction, and use strategies to promote spontaneity, initiative, and generalization, including incorporation of family members in the interventions. They focus on developmentally based learning targets and important foundational social learning skills like joint attention and imitation known to facilitate acquisition of language and other higher-level skills.

Because NDBIs enjoy a strong research base that substantiates their efficacy for improving meaningful outcomes in young children with ASD, it is critical to disseminate this message and share this record of results with the research communities and the public sector. The public sector definition of “applied behavior analysis” is oftentimes wrongly equated with a specific method of ABA, DTT, rather than being understood as an umbrella of empirically based practices that are built on operant learning procedures. The remarkable work of many researchers, working in parallel in different locations and publishing independent and converging results on a wide range of NBDI approaches, have created a new generation of early intervention models whose common features and efficacy may not yet be widely known to parents, clinicians, physicians, and to funding agencies.

Confusion about the actual definition of ABA, and its incorrect interpretation as massed or discrete trial teaching may lead referrers and funders, including health insurance companies, to mistakenly restrict coverage for autism treatment to only one type of ABA, DTT, thus denying the full range of effective intervention approaches based on ABA to consumers. The NDBIs described in this paper are efficacious treatments based firmly in ABA and supported by a large body of evidence. It can be solidly argued that funding that provides coverage of ABA treatment should cover NDBIs.

In order to reduce confusion, we urge intervention researchers when conducting research or providing treatment using an NDBI to explicitly state that the intervention under study or use is a Naturalistic Developmental Behavioral Intervention. Such consistent use of this term will help policy makers, families, researchers, physicians, and other treatment providers to understand where within the continuum of intervention practices a specific intervention exists. Future research should lead to interventions that are even more effective, efficient and individualized. Better understanding of (1) the active ingredients of these interventions, (2) fidelity of implementation needed for good outcomes in both research and community settings, and (3) the components that have the strongest effect on outcomes for subgroups of children are critical research goals as the next generation of studies is designed.

## Footnotes

1 Throughout this paper we use the term “behavioral” to describe treatment approaches that focus on learning and behavior changes without medications, whether or not the underlying approach is from the science of applied behavior analysis (ABA).
